# Multi-Objective Optimization Method for Signalized Intersections in Intelligent Traffic Network

**DOI:** 10.3390/s23146303

**Published:** 2023-07-11

**Authors:** Xinghui Zhang, Xiumei Fan, Shunyuan Yu, Axida Shan, Rui Men

**Affiliations:** 1 Department of Automation and Information Engineering, Xi’an University of Technology, Xi’an 710048, China; 1180311021@stu.xaut.edu.cn (X.Z.); axida@bttc.edu.cn (A.S.); menr@ldxy.edu.cn (R.M.); 2 College of Electronics and Information Engineering, Ankang University, Ankang 725000, China; ysywzhm@163.com; 3 School of Information Science and Technology, Baotou Teachers’ College, Baotou 014030, China

**Keywords:** intelligent transportation, signalized intersections, multi-objective optimization, NSGA-III, denoising autoencoder

## Abstract

Urban intersections are one of the most common sources of traffic congestion. Especially for multiple intersections, an appropriate control method should be able to regulate the traffic flow within the control area. The intersection signal-timing problem is crucial for ensuring efficient traffic operations, with the key issues being the determination of a traffic model and the design of an optimization algorithm. So, an optimization method for signalized intersections integrating a multi-objective model and an NSGAIII-DAE algorithm is established in this paper. Firstly, the multi-objective model is constructed including the usual signal control delay and traffic capacity indices. In addition, the conflict delay caused by right-turning vehicles crossing straight-going non-motor vehicles is considered and combined with the proposed algorithm, enabling the traffic model to better balance the traffic efficiency of intersections without adding infrastructure. Secondly, to address the challenges of diversity and convergence faced by the classic NSGA-III algorithm in solving traffic models with high-dimensional search spaces, a denoising autoencoder (DAE) is adopted to learn the compact representation of the original high-dimensional search space. Some genetic operations are performed in the compressed space and then mapped back to the original search space through the DAE. As a result, an appropriate balance between the local and global searching in an iteration can be achieved. To validate the proposed method, numerical experiments were conducted using actual traffic data from intersections in Jinzhou, China. The numerical results show that the signal control delay and conflict delay are significantly reduced compared with the existing algorithm, and the optimal reduction is 33.7% and 31.3%, respectively. The capacity value obtained by the proposed method in this paper is lower than that of the compared algorithm, but it is also 11.5% higher than that of the current scheme in this case. The comparisons and discussions demonstrate the effectiveness of the proposed method designed for improving the efficiency of signalized intersections.

## 1. Introduction

With increasing population and vehicles, traffic congestion becomes more and more serious in urban areas. Managing and mitigating traffic congestion is one of the great challenges for urban management [1]. Mitigating the impact of traffic congestion is mainly carried out from three aspects, namely, building more road infrastructure, promoting alternative modes of transportation, and managing traffic flows. Building more road infrastructure can be limited by various factors, including environmental (e.g., inappropriate terrain), economic (e.g., budget), and social factors, and promoting alternative modes of transportation is mainly a public policy issue. By factoring connected autonomous vehicles and unconnected human-driven vehicles, a number of platoon control techniques are proposed in the literature [2,3], which can improve vehicular mobility and vehicle-to vehicle communication. In addition, effective traffic signal control can also better manage traffic flows, which is an effective scientific and technological means to alleviate urban traffic congestion at appropriate cost [4]. Thus, how to improve the traffic efficiency of urban signalized intersections effectively has become increasingly important.

According to the current situation of urban traffic control systems, they can be divided into fixed-time control, actuated control, and adaptive signal control systems [5]. At present, most signalized intersections in developing countries such as China [6] and developed countries such as the United States [7] are equipped with fixed-time controllers and semi-actuated or actuated controllers. The aim of traffic control is to improve traffic efficiency. One of the most common ways is to control the traffic lights at intersections. The design of decision variables can be expressed as an optimization problem aiming at a traffic efficiency index, also known as an intersection signal-timing problem (ISTP). In general, traditional traffic signal control strategies based on mathematical approaches can provide many useful ideas and new methods for applications [8]. However, mathematical approaches have difficulties in providing solutions for ISTPs, which are generally complex and nonlinear. Instead, computational intelligence (CI) methods have been proposed [9].

Various CI approaches have been proposed for traffic signal control schemes to solve ISTPs. The key idea of CI is to simulate the intelligence of nature to a certain extent by using computing methods, including artificial neural networks, fuzzy systems and evolutionary computing (EC) algorithms. Each method is well developed and has many branches. In addition, CI adopts approaches such as swarm intelligence [10], reinforcement learning [11], game theory [12], etc. Each method is well developed and has many branches; we focus on the application of EC algorithms to solve the ISTP.

The remainder of the paper is organized as follows. Section 2 presents a brief explanation of related work. Section 3 describes the traffic environment of the study area and gives the current timing of each intersection and its traffic flow survey values. In Section 4, the ISTP is defined and formulated from basic symbols to optimization objectives. Section 5 introduces the basic principles of DAE and the specific ideas and steps for combining it with NSGA-III. Section 6 describes the test verification and analysis of results in detail. Finally, in Section 7, we summarize some highlighted points and the potential research directions of this article.

## 2. Related Work

As mentioned earlier, the purpose of traffic signal optimization is to improve the performance of traffic networks. Examples of optimization objectives include minimizing the delay time, maximizing traffic capacity, minimizing exhaust emissions, and so on. Rouphail et al. [13] employed a GA to optimize the traffic signal timing of nine intersections in Chicago, USA. As far as the queue length is concerned, due to the slow convergence behavior of GAs, further improvement can be made. Garcia et al. [14] proposed an optimization method based on a PSO algorithm to find a successful timing scheme by adjusting the initialization and decoding of individuals. The obtained solution makes the number of vehicles arriving at a destination and the total travel time achieve a quantitative improvement. In [15], a swarm intelligence algorithm called discrete harmony search was proposed to minimize the total delay of a traffic network.

In addition, optimizing multiple objectives simultaneously has attracted researchers’ attention. For example, Jia et al. [16] formulated a multi-objective model based on per capita delay, vehicle emissions, and intersection capacity. In their model, a PSO-based method enhanced with a difference operator and dynamic relaxation strategy is presented, and the effectiveness of the algorithm is verified by numerical experiments for a single intersection. Similar studies are also available in [17]. For the ISTP, there are mutual conflicts among the objectives to be optimized. To address this problem, multi-objective evolutionary algorithms (MOEAs) have been proposed. In [18], in order to maximize the throughput and minimize the average queuing rate at a single intersection with traffic oversaturation, an NSGA-II algorithm was proposed to optimize traffic signal timing. However, since the classical control parameters, such as signal cycle and splits, are not used, the algorithm is not easily applied in existing signal control systems. Zhang et al. optimized signal-timing plans by considering the saturation flow of an intersection, which indicates the total delay, traffic capacity, and emissions of a single intersection [19]. Under the framework of an NSGA-III, a constraint strategy is embedded to obtain a better signal-timing scheme. Li and Wang et al. [20] combined a GA algorithm with the Pareto criterion to optimize four indicators and propose an algorithm to help users select and realize the optimal design from the Pareto optimal solution set. The experimental results showed that the capacity, delay, safety, and overflow effect of nine intersections were improved. Zhao et al. [21] established a multi-objective signal-timing optimization model for average vehicle delay, slow traffic delay, parking times, and traffic capacity. To solve the established model, the Pareto dominance theory was integrated into a PSO algorithm. The experimental results showed that several indicators were improved at a single intersection.

Although these models and methods focus on many aspects of intersection signal control, some points can still be worth studying. First, it is reasonable to consider the interests of motor vehicles for the optimization of signalized intersections, and it is also worthwhile to design optimization objectives reasonably to minimize the impact on the original objectives when considering the interests of non-motor vehicles. Second, in the multi-objective models with multiple intersections, the dimensions of the decision variables that need to be optimized are relatively high. Based on these two aspects, it is necessary to study reasonable strategies to better solve the ISTP.

The issues outlined above call for novel research on traffic control models and MOEAs. (1) Traditional signal control models (TRRL, ARRB, and HCM [22]) are still used as the basis of modeling. The traffic efficiency indicators represented by these models can be adapted to different traffic conditions [23]. On this basis, we can establish a reasonable target model for the ISTP combined with the traffic environment of the studied area. (2) In recent years, many MOEAs have been tailored to solve the various optimization problems in real-world settings, e.g., network structure learning [24], reactive power dispatch [25], allocation support funds [26], relief distribution [27], and so forth. In addition, an AutoML approach using evolutionary algorithm has been proposed for EEG signal classification [28]. The [29], the authors adopted a MOSA algorithm to improve the supply chain for personal protection equipment during the COVID-19 pandemic. Also, an improved MOEA/D was proposed for project portfolio optimization [30]. The [31], the authors present a well-designed coding scheme and a problem-specific local search mechanism to improve the performance of an MOEA/D. In 2020, to solve many objective problems, Zi-Min Gu et al. [32] improved the ability of an NSGA-III by introducing a feedback model. In [33], two unsupervised neural networks were used to approximate the Pareto-optimal subspace for large-scale multi-objective optimization. In their work, the authors designed a support vector regression predictor, which makes individuals better for adapting to the environment in a dynamic multi-objective optimization problem [34]. These applications not only inherit the original algorithm frameworks and advantages but also add new strategies, including information from the newly generated individuals to solve the shortcomings of the original algorithms. These previous results also indicate that a new MOEA algorithm based on hybrid mechanisms can be effective. It is worth researchers’ attention to improve the search effect of the algorithm by combining different components or strategies.

In this study, through the application of traffic flow theory and the improvement of a non-dominated sorting genetic algorithm, the following methods have contributed to the optimization at signalized intersections: (1) In [35,36], the intersection performance was improved by adding infrastructure. Different to them, this article adds the conflict delay between right-turning vehicles and straight-going non-motor vehicles at intersections as one of the performance indicators to be optimized in a multi-objective model and quantitatively analyzes its impact on the performance of intersections in the studied area. (2) The proposed NSGAIII-DAE algorithm utilizes the ability of the NSGA-III framework [37] to deal with the multiple objective problems and the feature extraction capabilities of the DAE [38] deep learning model and demonstrates their combination to solve the optimization of the high-dimensional ISTP.

## 3. Study Scenario and Data Description

The data utilized in this study were collected from four signalized intersections in the city of Jinzhou, Liaoning Province, China. In the city of Jinzhou, some roads are divided by railways, so traffic congestion easily occurs on the main roads. Most intersections are not equipped with dedicated right-turn phases, and vehicles are vulnerable to non-motor vehicles.

**Figure 1:**
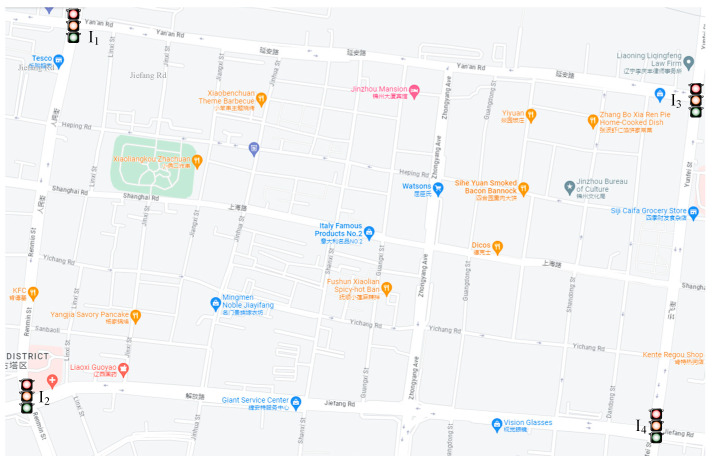
The layout of the regional traffic network in Jinzhou (from Google Street Maps).

Intersections I_1_ and I_3_, respectively, represent the intersections of Yan’an Road, Renmin Street, and Yunfei Street where the railway station is located. Intersections I_2_ and I_4_, respectively, represent the intersections of Jiefang Road, Renmin Street, and Yunfei Street. There are many commercial centers and government agencies near Jiefang Road (as shown in Figure 1). The proportion of non-motor vehicle travel is also high because of the continuous improvement of people’s awareness of green and low-carbon travel and the investment in shared-travel electric vehicles in recent years, so the mixing of motor vehicles and non-motor vehicles is serious. The traffic flow at these intersections is controlled and regulated by fixed/pre-timed signals. Two types of data are mainly acquired, i.e., traffic flow and signal cycle lengths, green splits, including the phasing sequence.

Table 1 shows the phasing scheme at four trunk intersections in the study area. I_1_ and I_2_ have four phases, I_3_ has six phases, and I_4_ has three phases. Taking phase 1 of intersection I_3_ as an example, “E-St” indicates that the east entrance is allowed to go straight during this phase. The traffic flow of the four trunk intersections is shown in Table 2. Motor vehicles in Table 2 mainly include cars, buses, and trucks, and non-motor vehicles mainly refer to electric motorcycles and bicycles. The saturation flow rate of vehicles is 300 veh/h in this study.

## 4. Definition and Formulation of the ISTP

Different optimization objectives can be adopted for the different actual requirements of the ISTP. Delay and capacity are the two most important criteria used for determining performance and the level of service of signalized intersections. Our scheme additionally considers the conflict delay between right-turning vehicles and straight-going non-motor vehicles because the traffic environment of Jinzhou City has the actual characteristics of the mixed traffic of motor vehicles and non-motor vehicles, and there is no right-turn phase set at intersections, in addition to the traffic capacity and signal control delay. To simplify the technical discussions, some key notations in the ISTP formulation are listed in Table 3.

- Vehicle control delay at signalized intersections

Here, the total average delay of vehicles is taken as the optimization objective of the trunk intersection, which can be written as follows:

(1) $$D_{v}=\sum_{k}^{N}\sum_{i}^{M}V_{i}\left(k\right)D_{i}\left(k\right)$$

where N is the maximum number of intersections, M is the maximum of phases at the intersection, $V_{i}\left(k\right)$ is the traffic volume of all lanes in the ith phase at the kth intersection, and $D_{i}\left(k\right)$ is the vehicle delay in the ith phase at the kth intersection. An ARRB model is a delay model of a signalized intersection suitable for variable demand conditions [39].$D_{i}\left(k\right)$ can be written as a function of $c\left(k\right)$, $S_{ij}\left(k\right)$, $x_{ij}\left(k\right)$, and $g_{i}\left(k\right)$. For more details, including constraints, please refer to [16].

- Conflict delay of right-turning vehicles

The dissipation process of non-motor vehicles gathered at an intersection after the signal light is released includes centralized dissipation and random dissipation. The crossing interval of right-turning motor vehicles appears randomly in the process of random dissipation, and right-turning vehicles can cross randomly, while there is almost no crossing interval in the process of centralized dissipation. At common signalized intersections, the arrival distribution of vehicles and non-motor vehicles generally follows a Poisson distribution, and the headway between non-motor vehicles follows a negative exponential distribution [40,41]. The total delay of right-turning vehicles crossing the straight-going non-motor vehicle flow at an intersection is expressed as follows:

(2) $$D_{v\text{\_}right}=\sum_{k}^{N}\sum_{i}^{M}\sum_{j}^{L}\left(\begin{array}{l} \frac{\left(1-e^{-\lambda_{ij}\left(k\right)u_{0}}\right)\left(\beta_{ij}\left(k\right)b_{ij}\left(k\right)+\beta_{ij}\left(k\right)a_{ij}\left(k\right)\right)}{\lambda_{ij}\left(k\right)e^{-\lambda_{ij}\left(k\right)u}\left(1-e^{-\lambda_{ij}\left(k\right)n_{queue}u_{0}}\right)}+\\ \frac{\beta_{ij}\left(k\right)a_{ij}{\left(k\right)}^{2}}{2}-u_{0}\left(\beta_{ij}\left(k\right)b_{ij}\left(k\right)+\beta_{ij}\left(k\right)a_{ij}\left(k\right)\right) \end{array}\right)$$

(3) $$\lambda_{ij}\left(k\right)=q_{ij\text{\_}sn}\left(k\right)\frac{e^{-q_{ij\text{\_}sn}\left(k\right)u/900}}{900}$$

(4) $$\beta_{ij}\left(k\right)=q_{ij\text{\_}r}\left(k\right)\frac{e^{-q_{ij\text{\_}r}\left(k\right)u/900}}{900}$$

where $\lambda_{ij}\left(k\right)$ and $\beta_{ij}\left(k\right)$ are the average arrival rates of the non-motor vehicles and right-turning motor vehicles, respectively. $q_{ij\text{\_}r}\left(k\right)$ is the traffic flow of right-turning vehicles at the jth entrance in the ith phase.

- Traffic capacity

The traffic capacity of a signalized intersection is estimated according to each entrance lane of the intersection. The capacity of an entrance lane in one direction is the sum of the capacity of each lane of the entrance lane. The capacity of an entrance lane is multiplied by the green signal ratio of its signal phase based on the saturation flow rate of the lane. The total capacity of multiple intersections is expressed as follows:

(5) $$CAP_{vehicle}=\sum_{k}^{N}\sum_{i}^{M}\sum_{j}^{L}S_{ij}\left(k\right)\left(\frac{g_{i}\left(k\right)}{c\left(k\right)}\right)$$

where $CAP_{vehicle}$ refers to the capacity of motor vehicles.

- Construction of the optimization objective model

The purpose of traffic control is to improve traffic efficiency. Generally, the traffic capacity and motor vehicle delay under signal control should be considered. According to the characteristics of the traffic environment in the studied intersection area, the delay caused by the conflict between the right-turning vehicle and straight-going non-motor vehicle flow is also considered. Our ISTP can be expressed by a mathematical formula as follows:

(6) $$f\left(X\right)=\min\left[-CAP_{vehicle}\left(X\right),D_{v}\left(X\right),D_{v\text{\_}right}\left(X\right)\right]$$

where the decision vector X is composed of the decision vectors of each intersection, specifically written as $X=\left(X_{1},\cdots ,X_{k},\cdots ,X_{N}\right)$, where $X_{k}$ represents the decision vector of intersection k. The specific elements of the decision vector of each intersection refer to the green time and signal cycle of the intersection, which are recorded as $X_{k}=\left(g_{1}\left(k\right),\cdots ,g_{L}\left(k\right),c\left(k\right)\right)$. $CAP_{vehicle}\left(X\right)$ is the total traffic capacity; $D_{v}\left(X\right)$ refers to the delay of motor vehicles under the control of traffic lights. Due to the large non-motor vehicle traffic flow in the study area, $D_{v\text{\_}right}\left(X\right)$ represents the delay of right-turning vehicles caused by straight-going non-motor vehicles. In the following sections of this article, it is referred to as the conflict delay. The negative expression symbol before $CAP_{vehicle}\left(X\right)$ does not mean that the value of $CAP_{vehicle}\left(X\right)$ is negative but indicates the Pareto relationship between $CAP_{vehicle}\left(X\right)$ and the other two goals.

## 5. Methodology

The NSGA-III algorithm is an advanced algorithm based on Pareto dominance and has shown good performance in multi-objective optimization problems. It uses the generated reference point information and niche technology to select a new parent population, which improves the selection pressure of the Pareto dominance relationship. However, when adopted in an ISTP, it faces a high-dimensional search space, and its optimization performance still has some room for further improvement. In order to solve the problems of feature extraction, large-scale calculation, slow function proficiency, and easy-to-fall-into local optimization, Hinton et al. proposed the training strategy of deep learning [42], and then some scholars proposed a denoising autoencoder (DAE) [38]. The main idea of the proposed NSGAIII-DAE algorithm is to train the deep learning network DAE using high-dimensional populations generated during the iteration process of the NSGAIII framework. Then, the trained DAE can be used to treat the Pareto solutions in low-dimensional space as a compact representation or approximation of the Pareto solutions in high-dimensional space. Next, there are two subsections. Firstly, the basic principles of the AE and DAE deep neural network models are explained. Secondly, the detailed steps for embedding the DAE deep neural networks in the NSGA-III are demonstrated.

### 5.1. General Denoising Autoencoder

**Figure 2:**
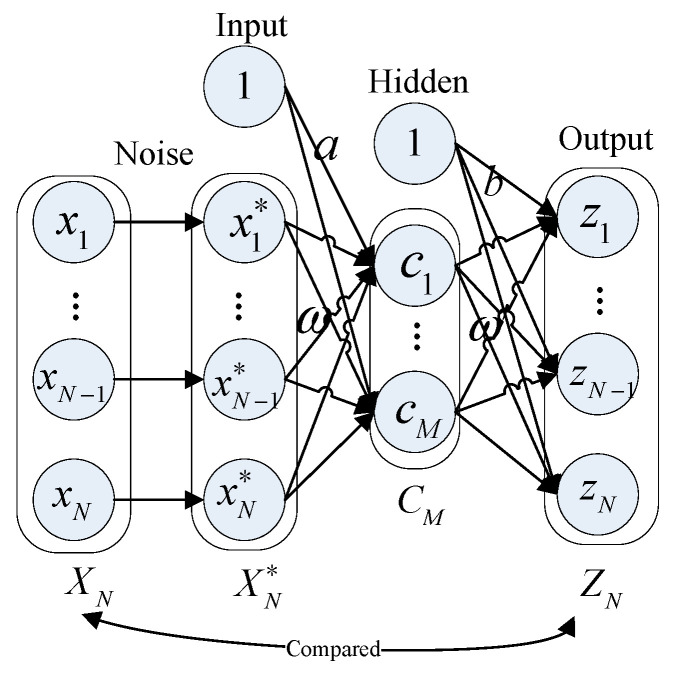
The network structure of a DAE and its training process.

A single automatic encoder (AE) is usually the basic structure of a deep artificial neural network for unsupervised feature extraction [43]. The DAE is a variant of an AE. The robustness is enhanced by adding noise with a specific distribution to the original input data. The DAE’s main structure is shown in Figure 2; it is a three-layer network model in structure. The network parameters between the input layer and the hidden layer are regarded as encoding operations, and the network parameters between the hidden layer and the output layer are decoding operations. $X_{N}=\left\{x_{1},x_{2},\cdots ,x_{N}\right\}$ represents the original data vector, and AE directly takes $X_{N}$ as the input of the model. The idea of the DAE is to add a noise signal to the original data $X_{N}$, and then take the generated noisy data $X_{N}^{*}$ as the input of the model. $C_{M}$ represents the hidden-layer vector, and $Z_{N}$ represents the output-layer vector. The output target of the DAE is equal to the original input data $X_{N}$, thus forming an ‘$X_{N}$-$C_{M}$-$Z_{N}$’-type neural network. Once the output $Z_{N}$ successfully copies the original data, it indicates that $C_{M}$ contains most of the information of the original data. The training process is represented by the following four steps:

Step 1: Add noise to input vector $X_{N}$ to obtain $X_{N}^{*}$. Generally, the types of noise added to a DAE are Gaussian noise, random noise, and mask noise. In this paper, random noise is used, which is expressed as follows:

(7) $$X_{N}^{*}=q\left(X_{N}\right)$$

where q is a random mapping.

Step 2: Encoding from the input layer to the hidden layer. The function of the encoding operation of the DAE is to extract the intermediate feature $C_{M}$ from the noise-added data $X_{N}^{*}$, and the value of each node $c_{j}$ is calculated as follows:

(8) $$c_{j}=\sigma \left(a_{j}+\sum_{i=1}^{N}x_{i}^{*}\omega_{ij}\right)$$

where $a_{j}$ represents the bias of the jth neuron of the hidden layer, $x_{i}^{*}$ is the ith element of $X_{N}^{*}$, and $\omega_{ij}$ represents the weight from the ith neuron of the input layer to the jth neuron of the hidden layer. σ refers to the activation function, and the specific formula is $\sigma \left(x\right)=1/\left(1+\exp\left(-x\right)\right)$.

Step 3: Decoding operation from the hidden layer to the output layer. This operation uses the input data $X_{N}$ as the target output and decodes the intermediate feature to obtain the reconstructed data $Z_{N}$, and the value of each node $Z_{i}$ is expressed as follows:

(9) $$z_{i}=\sigma \left(b_{i}+\sum_{j=1}^{M}c_{j}\omega_{ji}^{\prime }\right)$$

where $b_{i}$ represents the bias of the ith neuron of the output layer, $c_{j}$ is output value of the jth neuron in the hidden layer, and $\omega_{ji}^{\prime }$ represents the weight form the jth neuron in the hidden layer to the ith neuron of the output layer.

Step 4: Minimize the error between the input and reconstruction. The ultimate goal of training is to minimize the input $X_{N}$ and the reconstruction result $Z_{N}$ after encoding and decoding operations:

(10) $$L_{DAE}=\frac{1}{S}\left(\sum_{s=1}^{S}\left\|X_{N}-Z_{N}\right\|\right)$$

where $L_{DAE}$ represents the error between all reconstructed $Z_{N}$ and the original data $X_{N}$ in the sample set, and S is the number of sample sets.

### 5.2. Improving the NSGA-III Algorithm Embedded with a DAE

**Figure 3:**
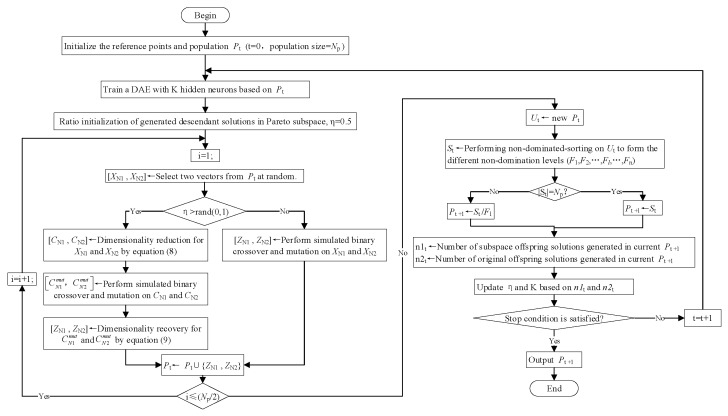
The main process of the NSGAIII-DAE.

Here, we describe in detail how the DAE is combined with the NSGA-III and explain the workflow of the NSGAIII-DAE algorithm. The main processing flow of the NSGAIII-DAE is shown in Figure 3, and the main steps are detailed as follows:

Step 1: Initialize population. NSGA-III is a genetic algorithm based on reference points. The initialization process includes generating a set of reference points, randomly initializing the population *P_t_* with size *N_p_*, and initializing the ideal points. The reference points are predefined according to Das [44]. *t* is the times of iterations, and *P_t_* is the population generated by the *tth* iteration. When t = 0, *P_t_* is referred to as the initial population.

Step 2: Train the DAE model. To make the DAE (as shown in Figure 2) compactly represent the decision variables $X_{N}$ or $Z_{N}$ of higher dimensions, the number of neurons (marked as M in Figure 2) in the hidden layer shall be set according to Formula 10, and its value shall be less than the number of neurons in the other two layers (marked as N in Figure 2). After using *P_t_* to train the DAE model by a gradient descent method, the model can encode data $X_{N}$ into $C_{N}$ and then decode it into $Z_{N}$ about equal to $X_{N}$.

Step 3: Generate offspring population $Z_{N1}$ and $Z_{N2}$. First, we need to randomly select two vectors each time from the current population P_t_ (two individuals randomly selected from $N_{p}$ individuals were marked as $X_{N1}$ and $X_{N2}$). The original NSGA-III algorithm generates the offspring population by using a crossover operator and mutation operator on the individuals in the parent population *P_t_*. At this time, each operation is directly carried out in the N dimensional space where the input vector is located. Whether each offspring solution is generated in the Pareto optimal subspace (dimension is M) or the original search space (dimension is N) can be determined by parameter η.

If η is greater than the random value of the [0, 1] interval, the offspring solution is generated in the Pareto optimal subspace. Specifically, the individuals $X_{N1}$ and $X_{N2}$ of the parent generation are compactly expressed as $C_{N1}$ and $C_{N2}$ according to Formula (8), and the crossover and mutation operations are performed to generate $C_{N1}^{mut}$ and $C_{N2}^{mut}$, which are decoded according to Formula (9) to restore the individuals $Z_{N1}$ and $Z_{N2}$ in the original N dimensional space. Otherwise, the descendant solution will be generated in the original search space without using the DAE. The vectors $X_{N1}$ and $X_{N2}$ in the current population *P_t_* directly cross and mutate in the original N dimensional space to generate $Z_{N1}$ and $Z_{N2}$.

Step 4: New generation population *P*_t+1_ is obtained. The current population *P*_t_ is updated by merging *P*_t_ with the offspring individuals $Z_{N1}$ and $Z_{N2}$ generated in each iteration. After all iterations are completed, the population, after removing the duplicate individuals in the population *P*_t_, is recorded as *U*_t_. In order to obtain the next generation population *P*_t+1_, we first need to perform non-dominated sorting on *U*_t_ and divide *U*_t_ into n different non-dominated ranks (*F*_1_, *F*_2_, …, *F_l_*, …, *F*_n_). From *F*_1_, we move a non-dominated level to the population *S*_t_ until |*S*_t_| ≥ $N_{p}$ (|*S*_t_| represents the number of individual solutions of the population). Suppose the level at this movement is *F_l_*, then *S*_t_ = (*F*_1_∪*F*_2_∪…∪*F_l_*). Then, it is necessary to observe whether |*S*_t_| is equal to $N_{p}$. If so, *S*_t_ directly acts as the next-generation population *P*_t+1_. Otherwise, we select ($N_{p}$-|*F*_1_∪*F*_2_∪…∪*F_l-1_*|) individuals from *F_l_* and merge them with (*F*_1_∪*F*_2_∪…∪*F_l-1_*) to form *P*_t+1_. The population size is still $N_{p}$.

Step 5: Update rules of hyper parameters related to the generation of offspring individuals. In the NSGAIII-DAE, there are two parameters related to the offspring, namely, the ratio η of the offspring solution generated in the Pareto optimal subspace and the size K of the hidden layer. Intuitively, parameter η should be adjusted dynamically and updated iteratively in the following way:

(11) $$\eta_{t+1}=0.5\times \left(\eta_{t}+\frac{n1_{t}}{n2_{t}}\right)$$

where $\eta_{t}$ represents the value of η at generation *t*, $\eta_{0}$ = 0.5, and $n_{1t}$ and $n_{2t}$ represent the number of successful subsolutions generated in the Pareto optimal subspace and the original search space of generation *t*, respectively. A successful offspring individual should be more likely to survive to the next generation. Therefore, the ratio $\left(n_{1t}/n_{2t}\right)$ and $\eta_{t}$ are added together to reflect the effectiveness of generating offspring individuals in the Pareto optimal subspace.

As for the hidden-layer size K, it should decrease with the population convergence to better balance exploration and exploitation. The K value is determined as follows:

(12) $$K=N-\left[\frac{2N}{3}\cdot \frac{t}{t_{\max}}\right]$$

where $t_{\max}$ is the maximum number of iterations, and [ ] is the rounding function.

Step 6: Determine whether the termination condition is met. For specific details, please refer to Deb’s literature [37]. If the termination condition is not satisfied, then *t* = *t* + 1, and we repeat Step 2. If it is satisfied, we output the final population.

## 6. Test Verification and Analysis of Results

As mentioned above, we have developed an evolutionary multi-objective algorithm, referred to as NSGAIII-DAE, which aims to find better traffic efficiency indicator values for an ISTP with high-dimensional decision variables. To verify the effectiveness of our method, the proposed algorithm and the designed optimization model are applied to four signalized intersections in the main urban area of Jinzhou City, China. Its layout is shown in Figure 1. The specific phase configuration of each intersection is shown in Table 1. The investigated traffic flow at intersections including motor vehicles and non-motor vehicles is shown in Table 2. In this paper, the ISTP (see Formula (6)) for these intersections is modeled as a multi-objective optimization problem. It mainly involves a signal control delay model, conflict delay model and traffic capacity model (see Formulas (1), (2), and (5), respectively). It is considered that there is no dedicated right-turn phase in our case study. Conflict delays due to conflicts between right-turning motor vehicles and non-motor vehicles going straight are common in the areas studied. Our ISTP considers this situation through Formula (2) discussed in Section 4. In addition, the decision variable dimensions in our ISTP are relatively high, with 21 dimensions. To this end, improvement ideas were discussed in Section 5.2, and the NSGAIII-DAE algorithm was proposed. In this section, numerical experiments are carried out from the following aspects to verify the performance of the proposed method. The optimization method was developed and solved in MATLAB (version R2020a). The experimental comparison and discussion conducted in this section are presented in the form of tables and charts.

- Analysis of Pareto solutions

The proposed NSGAIII-DAE algorithm learns the feature compression of solutions in high-dimensional spaces through a DAE, which enables the NSGA-III to perform genetic operations in low-dimensional spaces and then map back to high-dimensional spaces. By reducing the dimensions of the search space during the iteration process, the goal of improving performance is achieved. In order to verify the effectiveness of the NSGA-III algorithm embedded with a DAE, it is necessary to compare and analyze the Pareto solutions obtained by the proposed NSGAIII-DAE algorithm and the classical NSGA-III algorithm.

**Figure 4:**
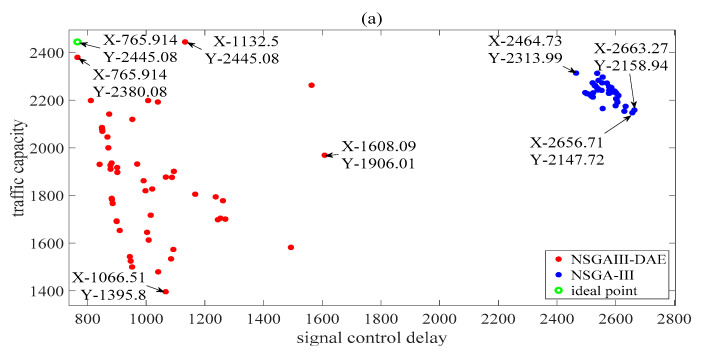

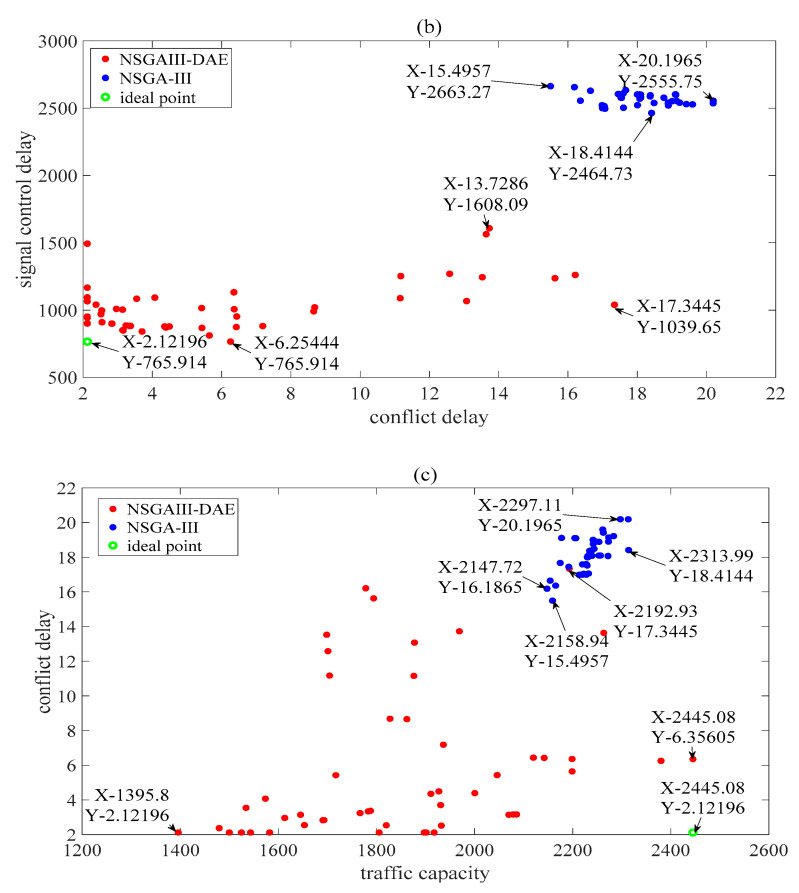
Pareto solutions between three objectives obtained with a 50 population size: (**a**) traffic capacity vs. signal control delay; (**b**) signal control delay vs. conflict delay; (**c**) conflict delay vs. traffic capacity.

**Figure 5:**
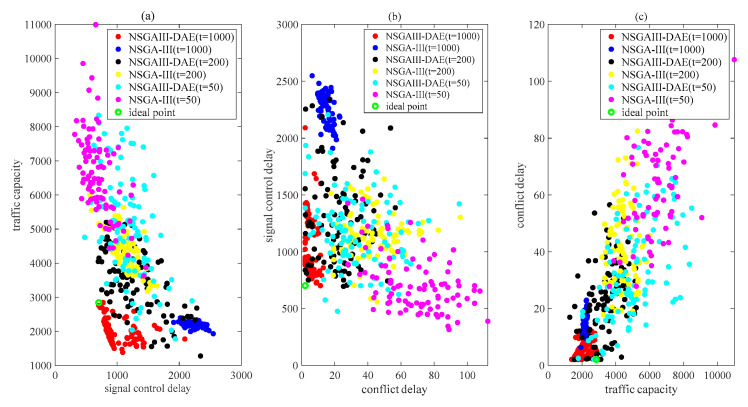
Pareto solutions between three objectives obtained with a 100 population size at tth iterations: (**a**) traffic capacity vs. signal control delay; (**b**) signal control delay vs. conflict delay; (**c**) conflict delay vs. traffic capacity.

In order to make a comparison, it is usually necessary to consider the determination of the appropriate population size and the number of iterations. For a 4-dimensional multi-objective problem at a single intersection [16], the authors set the population size to 50 and 100 and the number of iterations to 50 and 100 to test the proposed algorithm. In addition, a population number of 50 and an iteration number of 300 has been set up to solve the timing problem of adjacent intersections [45]. Considering the dimensions of the ISTP model, we set the population size to 50 and 100 and the number of iterations to 1000. The Pareto solutions obtained at the 1000th iteration before and after the introduction of the DAE are shown in Figure 4 and Figure 5, respectively. To facilitate the visual observation of the Pareto solutions involving three objectives obtained by the two algorithms, Figure 4 and Figure 5 each have three subgraphs, each displaying the values of each of the two performance indicators. In Figure 5, to further demonstrate the improvement in the algorithm with the number of iterations, the Pareto solutions obtained at the 50th and 200th iteration are also shown. In Figure 5, when the number of iterations is 50 and 200, it is easy to observe that the dots obtained after introducing the DAE are closer to the ideal points in its subgraphs, indicating that the performance of proposed NSGAIII-DAE algorithm is better. Based on the previous work on hyperparameter settings, we will continue to pay detailed attention to the performance of the algorithm at the 1000th iteration in Figure 4 and Figure 5. The explanations related to Figure 5, unless otherwise specified, refer to the situation at the 1000th iteration.

Considering that the convergence and diversity of the algorithm are quantitatively described in combination with Figure 4 and Figure 5, we adopt the Hyperarea (abbreviated as H) [46] and Pareto Spread (abbreviated as $D_{i}$) [47] as the performance indicators. Hyperarea (H) is a convergence indicator and Pareto Spread ($D_{i}$) is a diversity indicator. Let $v_{i}$ be the non-dominant solution point in $NDS_{known}$, and $a_{i}$ represent the high-dimensional space formed by the ideal point and $v_{i}$. $NDS_{known}$ represents the obtained approximate Pareto solution set, and $D_{i}$ represents the spread of the approximate Pareto solution set over the ith object space. H and $D_{i}$ can be explained as follows:

(13) $$H=\left\{\underset{i}{\cup }a_{i}\left|v_{i}\in NDS_{known}\right.\right\}$$

(14) $$D_{i}=\left|\max_{k=1}^{\left|NDS_{known}\right|}f_{i}\left(x_{k}\right)-\min_{k=1}^{\left|NDS_{known}\right|}f_{i}\left(x_{k}\right)\right|$$

For example, as shown in Figure 4a, the signal control delay values of all individual solutions obtained by the NSGAIII-DAE are better than those of the NSGA-III. Observing the traffic capacity indicator values in Figure 4a,c, although the proposed NSGAIII-DAE obtains a certain proportion of smaller traffic capacity values, the distribution range of capacity values is wider than that of the NSGA-III. Figure 4b shows the Pareto solutions of two delay performance indices. Except for a few red dots on the right side, the values of the two delay indicators corresponding to the vast majority of solutions obtained by the proposed NSGAIII-DAE are significantly better than those of the NSGA-III.

Figure 5 shows the Pareto solutions of the performance index values obtained by the two algorithms when the population size is 100. From each subgraph in Figure 5, it can be intuitively observed that the dots of the NSGA-III are more concentrated than those of the NSGAIII-DAE. However, for Figure 5a,b, at least some of the red dots are closer to the ideal point than all the blue dots. As shown in Figure 5c, the solutions obtained by the NSGA-III with better quality are almost surrounded by the solutions of the NSGAIII-DAE, but at the same time, it is obvious that some red points are closer to the ideal points.

In summary, it can be seen that the proposed NSGAIII-DAE has a more significant improvement in convergence or diversity compared to the NSGA-III. Figure 4 and Figure 5 provide an intuitive defense of this, and Table 4 provides a quantitative defense of this. Rows 2 to 7 of Table 4 list the D-values of the non-dominated solutions obtained before and after the introduction of the DAE by the NSGAIII (calculated according to Formula (14)). For the same optimization objective, the larger the pair of D-values to be compared, the wider the distribution range of the Pareto solution set on the objective. The last two columns of Table 4 list the H-value (calculated according to Formula (13)) of the non-dominated solution set obtained before and after the introduction of the DAE by the NSGAIII. The smaller the value is, the better the convergence performance is. In Table 4, most of the H values and all the D values show that the NSGAIII performs better after the introduction of the DAE. These values indicate that after introducing the DAE, populations of the same size can explore more promising spaces, which is conducive to better solving our ISTP.

- Influence of the conflict delay

Based on the characteristics of the traffic environment in the studied area, this article not only considers the signal control delay and the traffic capacity as optimization objectives but also includes the conflict delay based on the traffic flow of right-turning vehicles and straight-going non-motor vehicles. In order to test the impact of introducing conflict delay as an optimization objective in our ISTP on the overall performance indicator of intersections, experiments were designed, and the results were recorded, as shown in Table 5. The third column in Table 5 provides the performance indicator value corresponding to the current scheme. The fourth and fifth columns are the corresponding performance indicator values obtained after the algorithm optimization proposed in this article, and their results are significantly better than the current scheme. The difference is that for intersection I_1_, the fourth column uses the corresponding real traffic flow in Table 2, which is recorded as ‘actual’ in Table 5. The fifth column assumes that the traffic flow of straight-going non-motor vehicles and right-turning vehicles at intersection I_1_ has doubled and is recorded as ‘assumed’ in Table 5.

In addition to the two total delay and capacity results, the absolute percentage ratio (APR) of the results for the intersection where a sudden change in traffic occurred was also calculated in both cases. The formula for calculating APR is as follows:

(15) $$APR_{km}=\frac{A_{km}}{Total_{km}}\times 100\%$$

when k is taken as 1 or 2, it represents the corresponding ‘actual’ and ‘assumed’ cases. Take m as 1, 2, and 3 to represent the signal control delay value, traffic capacity value, and conflict delay value, respectively. ${Total}_{km}$ represents the absolute value of the mth indicator for all intersections under scenario k, while $A_{km}$ is the absolute value of the subintersection with sudden flow changes. For the ‘actual’ case, the ratios of the various indicator values of I_1_ to their total are 22.01% ($APR_{11}$), 29.10% ($APR_{12}$), and 60.45% ($APR_{13}$), respectively. In the case of “assumed”, the proportions of various indicators of I_1_ are 30.37% ($APR_{21}$), 15.59% ($APR_{22}$), and 16.09% ($APR_{23}$), respectively.

**Figure 6:**
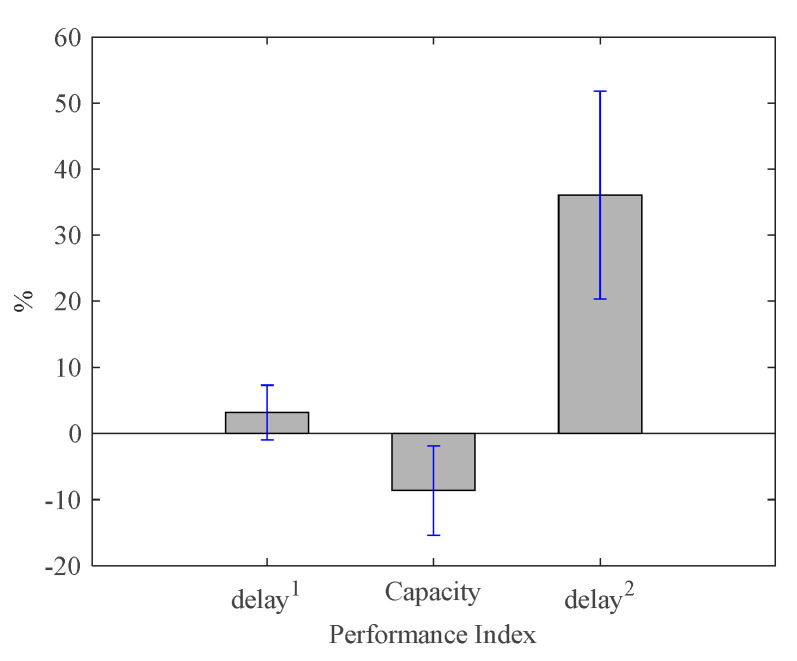
The average value and confidence interval of the APR before and after a sudden change in traffic volume at a single intersection. ‘delay^1^’ means signal control delay; ‘delay^2^’ means conflict delay.

Next, compare the changes in the APR values of the three indicators under the ‘actual’ and ‘assumed’ cases. Taking the delay^1^ of intersection I_1_ as an example, the APR value increased by 8.36%. Similarly, it can be seen that the APR value of capacity decreased by 13.51%, and the APR value of delay^2^ decreased by 44.36%. This indicates that when there is a significant increase in conflict delay at intersection I_1_, by reducing the delay^1^ indicator value by 8.35%, the capacity indicator by 13.53%, and the delay^2^ indicator by 43.41%, the overall performance of the intersections (I_Total_) in the region remains basically unchanged. In order to quantify the credibility of the experimental results, we conducted 16 experiments as described in Table 5. Figure 6 shows a columnar representation of the average change in APR values obtained from these 16 experiments with sudden changes in traffic flow and displays the 95% confidence intervals for the change in APR values of each of the three indicators, as shown in the blue line segment in Figure 6. These can help us reach the following conclusion: when there is a significant increasing trend of conflict delay at a certain intersection, the results obtained by our strategy increase the control delay of the intersection by about −0.97% to 7.31%, reduce traffic capacity by 1.84% to 15.43%, and increase conflict delay by 20.36% to 51.83%, keeping the overall performance index values of the four intersections basically unchanged.

- Comparison of performance indices with another algorithm

In this subsection, in order to test whether the NSGAIII-DAE is competitive in solving the ISTP, we compare the proposed algorithm with an existing algorithm. The HCNSGA-III [19] is a method to improve the NSGA-III by using a constraint processing strategy. Therefore, it is relatively reasonable to compare the proposed algorithm with the HCNSGA-III. In Table 6, the signal control delay, traffic capacity, and conflict delay of the intersections are listed, and the HCNSGA-III is verified and compared with the proposed NSGAIII-DAE. In addition to the two total delay and capacity results, the relative percentage deviation (RPD) between the performance index values obtained from each algorithm to be compared and the values obtained from the current scheme (CS) was calculated. The formula for calculating RPD is as follows:

(16) $$RPD\left(k\right)=\frac{R_{k}-R_{CS}}{R_{CS}}\times 100\%$$

where $R_{CS}$ is the result of the fixed signal timing currently in use, and $R_{k}$ is the result from the kth algorithm to be compared. Regarding the results, a better delay value corresponds to a smaller RPD value, while a better capacity value, corresponds to a larger RPD value.

Table 6 shows the optimal and average values obtained by the two algorithms, HCNSGA-III and NSGAIII-DAE, for the three optimization objectives of the ISTP and the corresponding RDP values. The bold font represents where the algorithm has the best result on the corresponding index. It can be seen from Table 6 that compared with the CS, the RPD value of the optimal value and average value of the NSGAIII-DAE method has an increase of 56.1% and 53.3%, respectively, in signal control delay, and an increase of 88.3% and 76.2%, respectively, in conflict delay, which is significantly better than the HCNSGA-III method. For the capacity index, compared to the CS method, its optimal value has also increased by 11.5%, and the average situation may reduce the capacity by 0.4%. However, the HCNSGA-III performs best in the capacity index. Through the analysis of the numerical results in Table 6, it can be concluded that the strategy proposed in this paper can significantly improve the signal control delay and conflict delay compared to the current scheme, while at least not reducing the traffic capacity.

**Figure 7:**
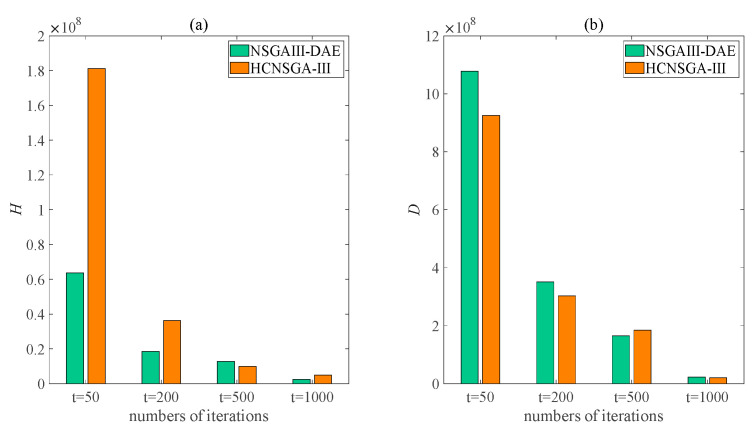
Comparison of the (**a**) H- and (**b**) D-values of the Pareto solution set obtained by the HCNSGA-III and NSGAIII-DAE algorithms at the 50th, 200th, 500th, and 1000th iterations.

To further verify the effectiveness of the proposed algorithm, Figure 7 presents more illustrative graphs for the H- and D-values obtained by the HCNSGA-III and NSGAIII-DAE algorithms under several different iterations. In addition to the 500th iteration in Figure 7a, it can be seen that the whole H-value of the proposed algorithm is smaller compared to the others. Similarly, it can be observed from Figure 7b that the D-value of the algorithm in this paper is relatively larger, except for in the 500th iteration case. Overall, for the evaluation indicators mentioned in Figure 7, the proposed NSGAIII-DAE also exhibits better diversity and convergence compared to the HCNSGA-III. Thus, it is evident from the results of the current study that the proposed algorithm is more capable of solving the ISTP.

## 7. Conclusions

This article studies the multi-objective optimization problem of signalized intersections and establishes a mathematical model for the ISTP based on the traffic environment of the studied area. In the proposed model, in addition to the usual signal control delay and traffic capacity, the conflict delay is specially considered as an optimization objective. An effective evolutionary algorithm based on the NSGA-III has been proposed to handle this high-dimensional model. In response to the challenges of diversity and convergence faced by the NSGA-III algorithm in solving the model presented in this paper, a DAE model is adopted to learn the high-dimensional space features for a dimensionality-reduction search. The algorithm was tested through actual cases, and the results showed that the proposed algorithm can obtain a better Pareto solution for the optimization objective within the reasonable number of iterations considered. In addition, the effectiveness of this algorithm was verified by comparison with the recent HCNSGA-III. In this work, the application of the proposed method will significantly improve the traffic conditions of urban road intersections. The combination of conflict delay issues and traffic control strategies can provide technical support for improving the service level of the entire road traffic system and promoting the flow regulation capacity of urban traffic.

Although the developed method exhibits good potential, there are still some limitations. In terms of the algorithm itself, we will try to apply the DAE model to the other improved MOEAs, and further study the effect of the model on a series of MOEAs. In practical traffic engineering applications, it may be necessary to use prior traffic information to reduce the search space for high-dimensional decision variables and combine traffic knowledge to study and determine the reasonable timing parameters. In addition, for further application in a vehicle–road collaborative environment and wider control range, using a distributed model based on edge intelligence [48] and a lightweight model based on a knowledge distillation framework [49] to design traffic signal control methods may be very promising. All of these are directions for our future efforts.

## Figures and Tables

**Table 1:** Current configuration of phase sequences at trunk intersections I_1_–I_4_.

	Phase Sequence					
	1	2	3	4	5	6
I_1_, I_2_	E ^1^&W ^2^-ST ^3^	E&W-L ^4^	S&N-ST	S&N-L	---	---
I_3_	E-ST	W-ST	W-R ^5^	W-L	S&N-ST	S&N-L
I_4_	E&W-ST	E&W-L	S&N-ST	---	---	---

^1^ east; ^2^ west; ^3^ straight; ^4^ left; ^5^ right.

**Table 2:** Investigation of the traffic flow at trunk intersections I_1_–I_4_.

No		Motor Vehicle Flow (veh/15 min)				Non-Motor Vehicle Flow (veh/15 min)			
		East	West	South	North	East	West	South	North
I_1_	straight	88	113	104	157	55	28	210	142
	left	24	4	57	127	7	10	16	20
	right	128	28	11	23	75	20	17	15
I_2_	straight	121	128	142	155	46	80	114	137
	left	101	55	63	60	18	35	78	49
	right	27	59	16	27	32	69	26	22
I_3_	straight	53	78	105	154	48	33	140	243
	left	32	69	25	73	46	37	13	59
	right	63	47	5	38	64	16	1	60
I_4_	straight	130	170	164	123	85	77	154	158
	left	37	45	59	29	21	16	28	43
	right	16	17	6	19	15	7	15	13

**Table 3:** Notations used in the intersection signal-timing problem.

Notation	Meaning
i	phase index at intersections, i=1,2,⋯,M;
j	approach index at intersections, j=1,2,⋯,L;
$\left(k\right)$	intersection index, k=1,2,⋯,N;
$D_{v}$	the total vehicle delay in a signal cycle;
$c\left(k\right)$	the signal cycle of the kth intersection;
$g_{i}\left(k\right)$	the effective green time of the ith phase;
$q_{ij}\left(k\right)$	the motor traffic volume of the jth approach at the ith phase;
$q_{ij\text{\_}sn}\left(k\right)$	the traffic volume of straight non-motor vehicles at the jth approach of the ith phase;
$S_{ij}\left(k\right)$	the lane saturation flow of the jth lane at the ith phase;
$x_{ij}\left(k\right)$	the traffic saturation of the jth lane at the ith phase;
u	The safe time interval, and the value here is 5 s;
$n_{\mathrm{queue}}$	The number of vehicles in a motor vehicle fleet that can be accommodated in a right-turn lane.
$u_{0}$	The minimum headway of a right-turning vehicle passing the conflict point, which is 2 s;
$b_{ij}\left(k\right)$	The duration of the random dissipation process of the jth approach of the ith phase, $b_{ij}\left(k\right)=g_{i}\left(k\right)/3$;
$a_{ij}\left(k\right)$	The duration of the centralized dissipation process of the jth approach of the ith phase, $a_{ij}\left(k\right)=g_{i}\left(k\right)/5$.

**Table 4:** The H - and D -values of the $NDS_{known}$ shown in each subgraph of Figure 4 and Figure 5. The best results in each of the two rows to be compared are bolded.

H or D	Figure 4a	Figure 4b	Figure 4c	Figure 5a	Figure 5b	Figure 5c
$D_{NSGA-III}$ (delay ^1^)	198.54	×	×	465.62	×	×
$D_{NSGAIII-DAE}$ (delay ^1^)	**842.17**	×	×	**924.94**	×	×
$D_{NSGA-III}$ (Capacity)	166.27	×	×	618.34	×	×
$D_{NSGAIII-DAE}$ (Capacity)	**1049.28**	×	×	**1388.86**	×	×
$D_{NSGA-III}$ (delay ^2^)	×	4.701	×	×	18.765	×
$D_{NSGAIII-DAE}$ (delay ^2^)	×	**15.223**	×	×	**30.851**	×
$H_{NSGA-III}$	5.231 × 10^5^	3.390 × 10^4^	4.798 × 10^3^	9.347 × 10^5^	1.043 × 10^4^	**9.003 × 10^3^**
$H_{NSGAIII-DAE}$	**3.008 × 10^5^**	**4.732 × 10^3^**	**4.270 × 10^3^**	**5.601 × 10^5^**	**7.451 × 10^3^**	1.228 × 10^4^

^1^ signal control delay; ^2^ conflict delay; ‘×’ indicates a value that does not need to be repeated, so it is not repeated here.

**Table 5:** Details of the results of a case study on a sudden change in traffic at intersection I_1_.

	PI ^4^	Current Scheme	The Proposed Method	
			Actual	Assumed
	delay ^1^	525.95	180.13	244.10
I_1_	capacity	524.00	704.00	400.00
	delay ^2^	5.48	2.14	0.93
	delay ^1^	471.54	263.24	214.94
I_2_	capacity	528.00	400.00	760.00
	delay ^2^	3.5900	0.61	0.61
	delay ^1^	379.14	160.78	209.09
I_3_	capacity	756.25	1015.00	515.00
	delay ^2^	4.59	0.47	0.98
	delay ^1^	393.08	214.38	135.75
I_4_	capacity	493.42	300	890.00
	delay ^2^	4.24	0.32	3.26
	delay ^1^	1769.71	818.53	803.88
I_total_^3^	capacity	2301.67	2419.00	2565.00
	delay ^2^	17.9	3.54	5.78

^1^ signal control delay; ^2^ conflict delay; ^3^ represents the four intersections within the area as a whole; ^4^ performance index.

**Table 6:** Comparisons of the HCNSGA-III and NSGAIII-DAE on three objectives.

PI ^3^	CS	HCNSGA-III		NSGAIII-DAE		RPD(%) HCNSGA-III		RPD(%) NSGAIII-DAE	
		Opt	Ave	Opt	Ave	Opt	Ave	Opt	Ave
delay ^1^	1769.7	1372.7	1889.2	776.2	826.3	−22.4	6.8	**−56.1**	**−53.3**
capacity	2301.7	2998.7	2698.9	2566.8	2292.8	**30.3**	**17.3**	11.5	−0.4
delay ^2^	17.9	7.7	13.7	2.1	4.26	−57.0	−23.5	**−88.3**	**−76.2**

^1^ signal control delay; ^2^ conflict delay; ^3^ performance index.

## Data Availability

All the data used to support the findings of this study are included within the article.
